# Improved postpartum care after a participatory facilitation intervention in Dar es Salaam, Tanzania: a mixed method evaluation

**DOI:** 10.1080/16549716.2017.1295697

**Published:** 2017-05-12

**Authors:** Eunice Pallangyo, Columba Mbekenga, Pia Olsson, Christine Rubertsson, Carina Källestål

**Affiliations:** ^a^ School of Nursing and Midwifery, Aga Khan University, Dar es Salaam, Tanzania; ^b^ International Maternal and Child Health (IMCH), Department of Women’s and Children’s Health, Uppsala University, Uppsala, Sweden; ^c^ School of Nursing, Department of Community Health, Muhimbili University of Health and Allied Sciences, Dar es Salaam, Tanzania

**Keywords:** Healthcare providers, postpartum care, quality, facilitation, Tanzania

## Abstract

**Background**: In order to improve the health and survival of mothers/newborns, the quality and attendance rates of postpartum care (PPC) must be increased, particularly in low-resource settings.

**Objective**: To describe outcomes of a collegial facilitation intervention to improve PPC in government-owned health institutions in a low-resource suburb in Dar es Salaam, Tanzania.

**Methods**: A before-and-after evaluation of an intervention and comparison group was conducted using mixed methods (focus group discussions, questionnaires, observations, interviews, and field-notes) at health institutions. Maternal and child health aiders, enrolled nurse midwives, registered nurse midwives, and medical and clinical officers participated. A collegial facilitation intervention was conducted and healthcare providers were organized in teams to improve PPC at their workplaces. Facilitators defined areas of improvement with colleagues and met regularly with a supervisor for support.

**Results**: The number of mothers visiting the institution for PPC increased in the intervention group. Some care actions were noted in more than 80% of the observations and mothers reported high satisfaction with care. In the comparison group, PPC continued to be next to non-existent. The healthcare providers’ knowledge increased in both groups but was higher in the intervention group. The *t*-test showed a significant difference in knowledge between the intervention and comparison groups and between before and after the intervention in both groups. The difference of differences for knowledge was 1.3. The providers perceived the intervention outcomes to include growing professional confidence/knowledge, improved PPC quality, and mothers’ positive response. The quality grading was based on the national guidelines and involved nine experts and showed that none of the providers reached the level of good quality of care.

**Conclusions**: The participatory facilitation intervention contributed to improved quality of PPC, healthcare providers’ knowledge and professional confidence, awareness of PPC among mothers, and increased PPC attendance.

## Background

This article reports the outcome of a collegial facilitation intervention project, ‘Improving postpartum care’ (IPPC), in a low-resource suburb in Dar es Salaam, Tanzania. Postpartum care (PPC) is neglected globally, particularly in sub-Saharan Africa, regardless of its potential to advance the health and survival of mothers/newborns [1]. In Tanzania, low PPC attendance at health institutions has been reported – 31% in 2010 [2] – despite a high maternal mortality rate (474/100,000 live births) and neonatal mortality rate (26/1000) [2]. However, about 75% of the children aged 12–23 months in Tanzania and 92% in Dar es Salaam are fully immunized [2], implying that mothers/newborns are in contact with health institutions but little attention is given to other PPC components.

Our baseline investigation in two low-resource suburbs of Dar es Salaam, Intervention Group (IG) and Comparison Group (CG), showed that few PPC consultations were conducted [3] despite healthcare providers’ (HCP) high level of knowledge and positive attitude towards PPC. The HCPs were aware of PPC being suboptimal, as well as its potential for improving mothers’/newborns’ health and the need for improvement [3]. A total of four PPC consultations are recommended for all mothers/newborns in Tanzania and should include physical and psychological assessment, and information and counselling related to breastfeeding, self-care, child-care, family planning, sexual health, and domestic violence [4].

### Conceptual framework

Quality of care is a complex and evolving phenomenon with three core concepts; structure, process, and outcome [5,6]. The recent World Health Organization (WHO) quality of care framework for maternal/newborn health [7] describes their connection as: the health system creates the ‘structure’ within which the care ‘process’, with its two interlinked dimensions, provision and experience of care, takes place and produces the ‘outcome’, which comprises person and health-centered key practices. To achieve good quality of care, the availability of competent and motivated human resources, essential physical resources, and good clinical skills are needed [7].

### Study rationale and aim

Low-quality and non-existent PPC may contribute to maternal/newborn ill-health and deaths, particularly in low-resource settings. Describing the outcomes of the facilitation intervention may contribute to alleviating the situation by answering the following questions: Has the intervention contributed to an improvement of the HCPs’ knowledge of and attitudes towards PPC? How do the HCPs perceive the outcomes of the intervention? How do mothers attending PPC perceive the quality of the care? How do experts define good and excellent quality of PPC? Do the HCPs deliver good-quality PPC?

Therefore, this study was designed with the aim of describing the outcomes of a participatory facilitation intervention to improve PPC in government-owned health institutions in a low-resource suburb in Dar es Salaam, Tanzania.

## Methods

We used a mixed methods approach with focus group discussions, questionnaires, observations, exit interviews, and field-notes to obtain a multifaceted description [8] of the outcomes of the intervention. Endline data were collected among HCP and mothers attending PPC at health institutions in the IG and CG in 2016. Baseline data were collected in the IG and CG in 2015, before the intervention, and were used for comparisons.

### Study setting

This study was conducted at government dispensaries, health centres, and hospitals making up both the IG and CG in Dar es Salaam, Tanzania. Tanzania is an East African low-income country where 28% of the population live below the national poverty line [9]. Dar es Salaam is its largest city and has about 4.4 million inhabitants in its 3 suburbs (1.2 million in the IG and 1.4 million in the CG) [2]. The low-resource suburbs are characterized by mushrooming squatter settlements, unemployment, poverty, poor sanitation, and ill-health [2].

Governmental institutional health services in Tanzania are delivered at dispensaries, health centres, and district, regional, and consultant hospitals. The IG has 2 hospitals (1 referral), 1 health centre, and 28 dispensaries, while the CG has 2 hospitals (1 referral), 1 health centre, and 26 dispensaries. All levels have reproductive and child health (RCH) units that mostly provide services in antenatal care, HIV/AIDS, under-five care, family planning, vaccinations, and PPC. Most of the HCPs caring for mothers/newborns at the RCH units have training in midwifery.

### Intervention

To promote PPC we designed a participatory facilitation intervention with HCPs at health institutions. The design of the intervention was inspired by the Promoting Action on Research Implementation in Health Services (PARIHS) framework [10] and the promising results of similar facilitation projects in improving maternal/newborn health in Vietnam [11], India [12], and Nepal [13]. Facilitation was the strategy used to activate the HCP and is defined as the process where a facilitator helps a group to achieve its purpose by promoting team dynamics and the active involvement of all the group members [10].

Six facilitators were trained to facilitate their colleagues in improving PPC at three to five health institutions and they were supervised by a principal investigator. Facilitators’ training included facilitation skills, supervision, national PPC guidelines, baseline results, and updates about national and international research. The HCPs determined the priorities and methods for improving PPC in their workplaces.

### Data collection

We planned to collect data from government-owned institutions in the IG and CG in the suburbs.

Focus group discussions (*n *= 5) were conducted with HCPs (*n *= 38 out of 99) in the IG to grasp their perceptions of the intervention outcomes. Institutions with six or more HCPs working with PPC were selected to enable big enough groups for vivid discussions that generated rich data. Two dispensaries, one health centre, and two hospitals were included. The HCP had various professions (RNM [Registered Nurse Midwife] = 16, ENM [Enrolled Nurse Midwife] = 9, TNM [Trained Nurse Midwife] = 5, MO/CO [Medical officer/Clinical officer] = 8), 28 were women and 10 men, 12 worked at dispensaries, 9 at health centers, and 17 at hospitals. Oral and written information about the aim and the procedures of the study, and that participation was voluntary, were given to the prospective participants. Two of the invited HCPs declined participation due to time constraints. The participants decided on the place, day, and time for the discussion to maximize participation rates. All discussions were held at their home institutions after working hours to avoid interruptions with services and where privacy was possible. The participants received compensation for use of alternative and late transport home caused by their participation. The groups had 6–10 participants, were audio-recorded, held in Swahili, the national language, and moderated by CM (co-researcher) who was unknown to the participants. The moderator introduced these topics to the participants: *How did you perceive the IPPC intervention? What were the successes? What challenges were there? What led to success? What are your suggestions for sustainability and improvement of the intervention?* The sessions lasted from 45 to 80 minutes and the participants were actively engaged in the discussions, some more than others. We here present the results that relate to the outcomes while the findings about the process of the intervention will be presented elsewhere.

A questionnaire was used to assess knowledge and attitudes about PPC among all the HCPs who were at work during the data collection period in the IG and CG. It comprised 28 items and was modified from Eriksson et al.’s [11] questionnaire, which has been used previously in Vietnam and in the baseline study of this IPPC project. This endline study involved seven health institutions which did not participate in the baseline because HCPs were unavailable during data collection, but these institutions did participate in the intervention.

Observations of PPC were conducted at all levels of institutions to assess PPC practice among HCPs at 3–7 days after childbirth. The observation checklist was developed from the Tanzanian PPC guidelines [4], with additions from the WHO recommendations on PPC [14] and from research reports [15–17] to assess conducted care items as an estimate of the quality of care. This checklist included items on history-taking, and physical and psychosocial assessment of the mother/newborn. We planned to conduct 10 observations at each institution. However, more observations were completed in the IG at the hospitals and a health centre where mothers’ attendance was highest while fewer than 10 mothers were obtained for observation on the data collection date in some dispensaries. PPC activities in the CG were minimal across all levels of institutions.

To define quality of PPC, we consulted nine Tanzanian experts in RCH (clinically and academically active midwives and obstetricians), asking them to suggest which items in the observation list were to be included in PPC for the care to be excellent or good, and which ones were not needed for good quality. After the first consultation, to which five experts responded, the researchers reviewed the feedback, discussed, and developed a consensus, which led to developing a definition of good-quality PPC. The definition, using the checklist, was presented to all nine experts, asking them to confirm the definition or to suggest changes. Two experts out of the three who answered approved the definition. The third expert, who did not approve, wanted all items (*n *= 59) to be included as indicators of good quality.

To explore mothers’ views on PPC, exit interviews were conducted immediately after observation of their PPC consultations using an interview guide with four items, namely: *satisfaction with services, providers’ skills, duration of the visit,*and *suggestions for improving PPC*.

To describe the quality of PPC at institutions, field-notes were written by the research assistants after data collection at each institution based on a guide with the following topics: presence and use of national PPC guidelines; staff and space allocated for PPC consultations; and general impressions of the quality of PPC.

All data collection, except the focus group discussions, was conducted by research assistants (*n *= 6) who are university graduates and who work at a non-governmental organization research institute in Dar es Salaam. They were trained for three days about data collection, research tools, participants’ rights, and the obtaining of consent. As a part of the training, a reproducibility test [18] on the observations was completed, where all research assistants observed the same 10 PPC consultations and reproducibility was rated as very good, fair to good, and poor.

### Data analysis

The audio-recordings from the focus group discussions were transcribed to text in Kiswahili prior to the analysis and thereafter translated into English to enable the participation of non-Kiswahili-speaking authors. Thematic content analysis [19] was used to describe the themes within the outcomes of the intervention from the focus group discussions and field-notes. The analysis consisted of repeated readings of the texts; identifying, comparing, and contrasting; and describing the themes in the data [19].

We used R commander [20] for statistical analysis of the data obtained from the questionnaires, observations, and exit interviews. Outcome variables were observed PPC, women’s perceptions of PPC, and HCPs’ knowledge of and attitudes towards PPC. The frequencies of the variables were calculated for each group and were compared using Chi-square tests. The comparison between the baseline and endline evaluation was conducted using Student’s paired *t*-tests, and between the IG and CG with an independent *t*-test. A multiway ANOVA was used to find the difference in quality score between health institutions. Statistical significance was set at 5%. For the comparisons, only health institutions that participated in both data collections were included (*n *= 162).

## Results

Data were collected from 51 out of a total of 60 health institutions in the IG (*n *= 26) and the CG (*n *= 25). Nine institutions were excluded from the study (6 from CG and 3 from IG) as they did not provide RCH services (*n *= 8) or the HCPs were absent (*n *= 1). The distribution of participants in the individual data collection methods per group at baseline and endline is given in Table 1.

In total, 174 (out of about 200 employed) HCPs who were at work during the data collection period completed the knowledge and attitude questionnaire. The background factors showed no significant differences in relation to gender, age, education, professional groups, and levels of institution between the IG and CG (Table 2), which was also the case in the baseline survey.

**Table 1.** The number of participants in the individual data collections.

	Intervention group		Comparison group	
Method	Baseline	Endline	Baseline	Endline
Focus group discussions (HCP)	29	38	26	0
Questionnaire (HCP)	89	99	60	75
Observation (HCP)	13	213	12	62
Exit interview (mothers)	20	116	18	38
Field-notes (institutions)	13	26	12	25

**Table 2.** Frequency distribution and background factors of PPC providers.

Variable		Providers (*N *= 174)	IG (*n *= 99)	CG (*n *= 75)	*p*-value^a^
Gender	Women	162	92 (93%)	70 (92%)	0.9
	Men	12	7 (7%)	5 (8%)	
Age	20–30	47	28 (60%)	19 (40%)	0.5
	31–40	58	36 (62%)	22 (38%)	
	41–50	43	21 (49%)	22 (51%)	
	51–60	26	14 (54%)	12 (46%)	
Profession^b^	MCHA	24	11 (11%)	13 (17%)	0.1
	RNM	107	68 (69%)	39 (52%)	
	MO/CO	6	2 (2%)	4 (5%)	
	ENM	37	18 (18%)	19 (26%)	
Institution	Dispensary	132	76 (77%)	56 (75%)	0.8
	Health centre	15	9 (9%)	6 (8%)	
	Hospital	27	14 (14%)	13 (17%)	

Notes: ^a^Chi-square test.

^b^MCHA – Maternal and Child Health Aider with 1 year of training in midwifery. ENM – Enrolled Nurse Midwife with 2 years of training in nursing and midwifery. RNM – Registered Nurse Midwife with 3 years of training in nursing and midwifery. MO/CO – Medical officer and Clinical officer with 5 and 3 years of training in medicine, respectively.

The analysis of the focus group discussions resulted in the following themes: improved professional confidence and knowledge, improved PPC, and the postpartum mothers’ positive response. They are described next and illustrated by quotes.

### Improved professional confidence and knowledge

The HCPs in the focus group discussions claimed that the intervention had enhanced their professional confidence, knowledge, and collaboration between HCPs within and between institutions. The HCPs now provide PPC more in accordance with guidelines and with more agreed state-friendly attitudes and language to mothers and fathers. They stated that they are highly motivated to provide PPC and that they more often act proactively and engage in creative solutions to improve the care. The HCPs expressed that their trust in their ability to provide quality PPC is enhanced:

We had this doctor (…) who used to teach about breastfeeding. (…) When you hear he was coming – you wish to run away, because you don’t know what to show him. (…) The thing is he used to teach things which we could not practice. (…) But *today*, if he comes in, I am confident that I can even tell him: ‘Sit over there and let me show you how I should orient a mother on proper breastfeeding.’ (Focus group discussions, health centre)

**Figure 1.**
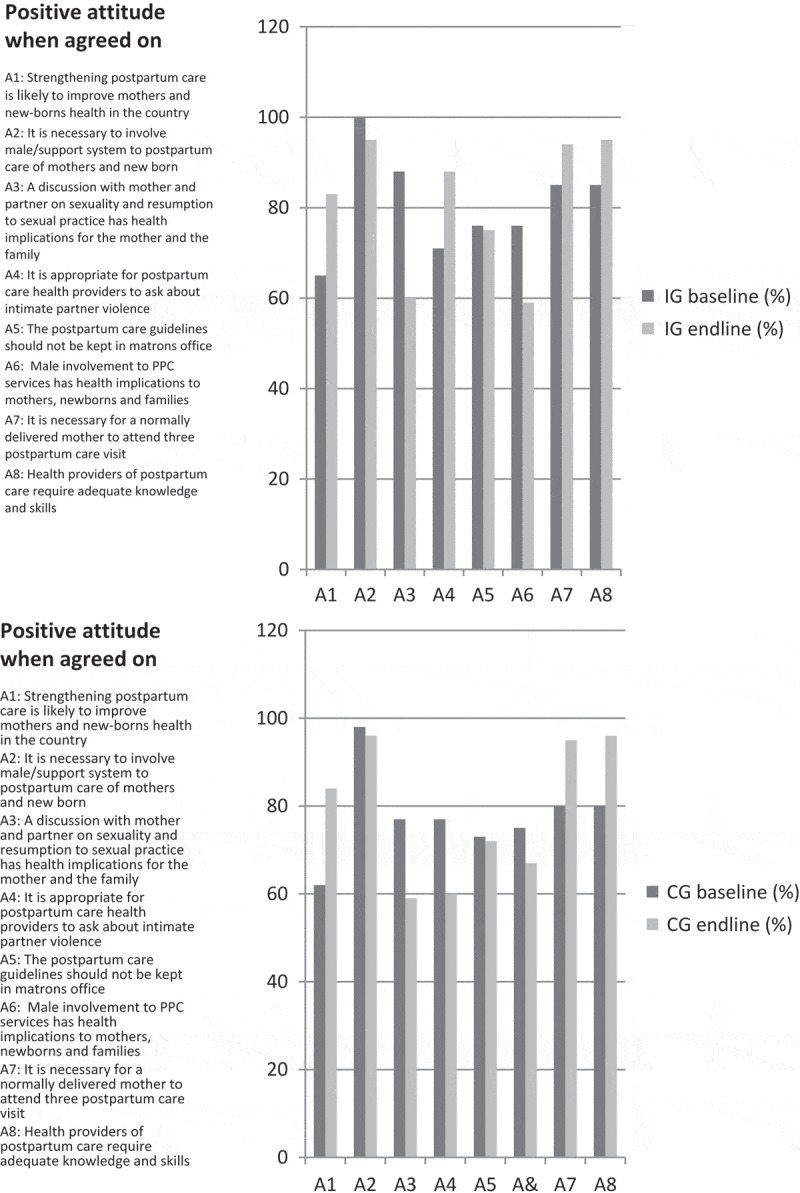
Distribution of the items in knowledge at baseline and endline in the two groups.

**Figure 2.**
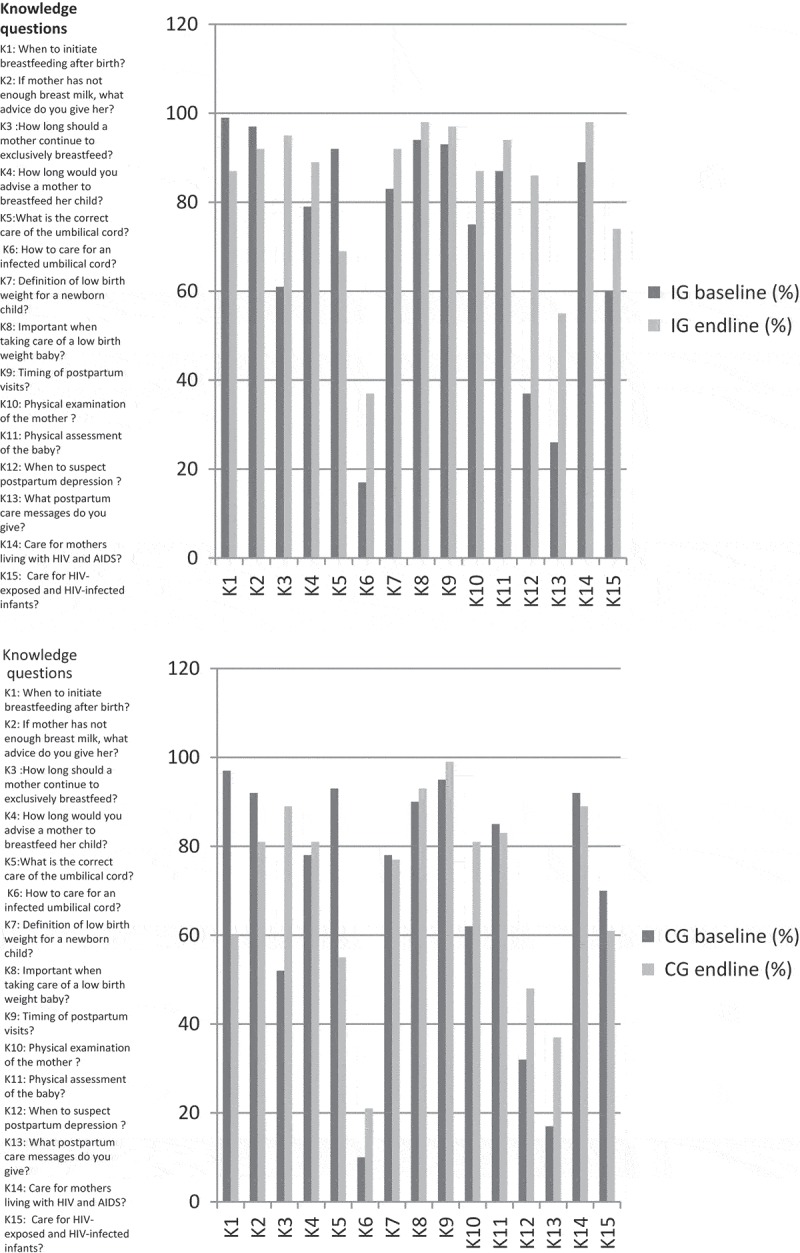
Distribution of the items in attitudes at baseline and endline in the two groups.

The analysis of the questionnaires showed that the HCPs’ knowledge of PPC increased after the intervention while the already relatively positive attitudes towards PPC remained. The proportions of positive responses to the items in knowledge (K1–K15) and attitudes (A1–A8) assessed at baseline and endline in the IG and CG are shown in Figures 1 and 2. The knowledge increased in K6, K12, and K13 in both the IG and CG but to a higher extent in the IG. The attitudes show no major difference between baseline and endline in both the IG and CG. The total scores were 15 and 8 for knowledge and attitudes, respectively.

The mean scores in knowledge in the IG were 9.4 (SD 1.3) at baseline and 12.5 (SD 2.1) at endline. The mean scores for the CG were 8.8 (SD 1.6) at baseline and 10.4 (SD 2.0) at endline. The *t*-test showed a difference in knowledge between the IG and CG at endline (*p *= 1.063e^−08^) and at baseline (*p*-value = 0.018), and a difference in knowledge before and after in the IG (*p*-value = 2.2e^−16^) and in the CG (*p*-value = 6.924e^−06^). The difference in differences for knowledge was 1.3.

The mean scores for attitude in the IG were 6.5 (SD 1.7) at baseline and 6.5 (SD 1.1) at endline, while for the CG they were 6.2 (SD 2.1) at baseline and 6.4 (SD 1.2) at endline. The *t*-test of the differences between the IG and CG on attitude at baseline and endline, respectively, showed no differences (*p*-values = 0.429 and 0.749). The difference between baseline and endline for attitudes in the two groups was not significant (*p*-values = 0.954 and 0.513), and the difference in differences was negative (−0.19).

### Improved PPC

In the focus group discussions the HCPs gave several examples of improved PPC quality as an outcome of the IPPC intervention. Analyses of field-notes (*N *= 51: IG *n *= 26, CG *n *= 25) also indicate the improvement of physical resources (Table 3). Some institutions had succeeded better than others. The improvements included organization, physical resources (Table 3), and provision of care, including the prevention and treatment of the health problems of mothers/newborns and increased partner involvement:

**Table 3.** Summary of physical resources in the IG and CG from analysis of field-notes.

Institution	PPCguideline	PPC space	PPC staff	Baby cot	Weigh scale	Blood pressure machine	Examination bed	Thermometer
IG	26	12	26	3	8	4	11	6
CG	0	4	0	0	0	0	2	4

Before we started the intervention one year ago we had PPC service but it was not done the way we are doing now. It was not seriously considered and most of the time we used to receive clients delivered on scissor (caesarean section), just for stitch removal. (…) After the intervention there are differences in how we deal with it, because we assess the mother and a newborn as well and give them advice. (Focus group discussions, hospital)

### Observations of PPC consultations

Observation was conducted at all levels of institutions (*N *= 275: IG *n *= 213, CG *n *= 62). The overall endline results show improvement in PPC in the IG (*n *= 213) (see Appendix 1) with no change in the CG (*n *= 62). The reproducibility test of research assistants was rated as follows: 44 items, very good; and 15 items, fair to good. The items on assessing mental health had particularly poor reproducibility.

More than 80% of the observed consultations included: greetings and showing interest; asking mothers if the baby had any problems; checking the baby for breathing and breastfeeding; counselling the mother for complications readiness and danger signs; providing counselling about danger signs for the newborns and what to do if the baby shows any danger signs; informing mother about next visit; and documenting these findings.

Less than 50% of the observations covered the following elements: encouraging the mothers to bring an accompanying person if they preferred; checking mothers’ records on rapid plasma reagin (RPR) and HIV tests; washing hands; the taking of vital signs; assessing bladder function; asking about history of past and perinatal mental health; counselling about malaria prevention, safer sex and sexually transmitted infections, and sexuality and sexual practice resumption; and voluntary testing of HIV. All other items were conducted in between 50% and 79% of the observations.

Differences in the care items performed between types of institutions were found. More items were performed at health centres and hospitals than at dispensaries. However, only 20 mothers were observed in the health centres and 30 in the hospitals, while 161 mothers were observed in dispensaries. In the CG, 5 mothers were observed in the health centre, 14 in the hospitals, and 43 mothers were observed in dispensaries.

The consolidated RCH experts’ opinion was that 46 out of 59 items of the observation checklist of PPC were required for judging the quality as good. In addition, the remaining 13 items were needed for PPC to be excellent. None of the items were judged by the experts as not needed. The distribution of items indicating ‘good’ and ‘excellent’ quality is shown in Appendix 2.

Evaluation of the observations of the PPC visits according to the experts’ suggestions shows that none of the providers reached excellent or good quality and the mean score achieved was 27.7 (SD 8.5) with a range of 7–43. The mean scores for types of institutions were as follows: dispensaries, 25.2 (SD 8.0); health centres, 38.1 (SD 2.8); and hospitals, 34.4 (SD 4.1). The difference between the three types of facilities was significant (*p*-value = 2.856^–16^). The mean duration of the PPC was 17.4 minutes (SD 9.7) per mother and newborn with a range of 4–58 minutes. The PPC activities in the CG were low with mainly BCG immunization of the newborn conducted at the under-five clinics.

### The postpartum mothers’ positive response

The HCPs participating in the focus group discussions enthusiastically reported about the increased number of postpartum mothers and their partners now attending PPC. At the dispensaries, the increase of mothers attending PPC was less prominent. However, at the referral facilities, the high number of mothers turning up for PPC could be overwhelming:

The number of mothers who are coming for postpartum has gone high. We receive between 15 up to 16. Yes, that is the number we receive on daily basis. That means for a month we receive between 150 up to 200. (Focus group discussions, hospital)

Mothers are described as appreciating PPC and are spreading the news, which reinforces the message to mothers, fathers, and the community that PPC visits at institutions are now recommended:

I have observed great awareness among community members, because even men are accompanying their wives to the PPC clinic. (…) This is new, previously you could never see that but now fathers are motivated. (Focus group discussions, hospital)

The exit interviews (*N *= 154: IG *n *= 116, CG *n *= 38) show that mothers are satisfied or very satisfied with HCP interpersonal relationship (IG *n *= 122, 99.19%; CG *n *= 34, 89.47%), duration of care (IG *n *= 108, 87.81%; CG *n *= 23, 60.53%), health providers’ skills (IG *n *= 117, 95.12%; CG *n *= 29, 76.32%), and general care provided to them and their newborns (IG *n *= 119, 96.75%; CG *n *= 34, 89.48%). The open answers in the exit interview show that the most appreciated care in the IG was the examination conducted with mothers/newborns, and the health education about PPC provided to mothers/newborns, and in the CG, it was the immunization of the newborns. However, mothers in both suburbs shared concerns about the length of time spent waiting for these services in a small waiting area and the bad language used by some HCPs. The mothers suggested the establishment of PPC rooms with privacy and enough space, increased numbers of health providers, and reduced waiting time. These were also areas for improvement suggested by HCPs in the focus group discussions.

## Discussion

The outcomes of this collegial facilitation intervention in these Tanzanian government-owned health institutions include: improved PPC attendance and quality of care; increased HCP knowledge and professional confidence; and increased PPC awareness among mothers. In the CG the PPC remained close to non-existent, as at baseline. These outcomes will be discussed using the concepts of the WHO quality of care framework [7]: human resources; infrastructure, equipment, and supplies; and clinical practice.

### Human resources

Competent and motivated human resources are important components in the improvement of quality of care [7]. The IPPC intervention improved HCP knowledge and skills, professional confidence, and motivation through training, teamwork, interactions, and collegial support. In Vietnam, the success in improving neonatal survival involved teamwork [11] and participants considered training and interactions as ways in which they acquired knowledge [21]. Training and HCP mentorship also improved quality of maternal health in Uganda and Zambia [22]. Promoting environments where HCPs will continuously interact and learn from colleagues may contribute to PPC improvement.

Baseline findings indicated positive HCP attitudes towards PPC; hence the minimal change in attitudes reported in the present study is not surprising. A remarkable change in attitudes would require more than a one-year intervention.

Despite the improvement in PPC in the present study, none of the HCPs met the definition of good or excellent quality as per the experts’ quality score. In Uganda and Zambia, the knowledge of HCPs also increased significantly after training and support, but the scores were far from the recommended levels [22]. Contributing factors to this could be the feasibility of the guidelines and criteria, the competency of the HCPs, and shortage of staff. The criteria used to define quality of PPC in the present study depart from the recommendations of the national PPC guidelines [4], which are detailed and difficult to fully apply in clinical practice, especially with scarce human resources. The criteria for good quality used in this study must be revised. For the guideline to really support how the HCPs work, we suggest that a revised PPC guideline should be more focused and have agreed criteria for good quality that are feasible for clinical practice and future evaluations.

The HCP shortage is a devastating obstacle for providing quality care in the study area, as in several low- and middle-income countries (LMIC) [23,24]. Nurses in 12 European countries reported skipping important tasks because of shortages of nurses [25]. Therefore, the HCPs may be competent and yet fail to fulfil the expectations of care due to staff shortages.

### Infrastructure, equipment, and supplies

The present study reports progress in securing space, equipment, and supplies for PPC in the IG. However, some spaces are small, less ventilated, and sparsely equipped. Resource deficiencies for healthcare services that negatively affect quality of care are widely reported in Tanzania [24] and in other LMICs. The low quality of care is anticipated as PPC consultations increase, particularly in a context where HCPs and infrastructure are deficient. Similar trends have been reported in other parts of Tanzania, Ethiopia, and Uganda [26]. A systematic review of maternal care interventions in LMICs also indicated a weak association between increased uptake of maternal health services and health outcome measures which is linked to quality and effectiveness of care [27]. There is evidence that investment in resources improves care; for instance, when the programme was complemented with investment in infrastructure and human resources in Uganda, HCPs’ confidence in providing care and mothers’ satisfaction were found to be higher than in the Zambian intervention [22]. The initiation of, and increase in, the PPC consultations reported in the present study are positive outcomes of the IPPC intervention, and an important step towards improving maternal/newborn health. However, strengthening infrastructures, equipment, and supplies is crucial in sustaining and extending the improvement.

### Improved clinical practice

In this intervention the quality of PPC practices improved in the IG and HCPs adhered more to the guidelines than before. The improved clinical practices were well received by the postpartum mothers. At baseline, none of the institutions possessed this guideline. Guidelines are useful for translating evidence into practice [28] and facilitate care provision effectively [29]. However, improvement of clinical practice requires high-quality guidelines [29]. The Tanzanian PPC guideline was developed in 2011 utilizing the WHO recommendations [14]. However, the guidelines lack any guidance in relation to mental health problems, sexuality, violence, and referral plans.

The WHO guidelines lack efficient, structured, and detailed plans for implementation [29]. The update of the existing Tanzanian PPC guidelines and the establishment of strategies to ensure the guidelines’ utilization in health institutions are of the utmost importance for improving PPC practices.

### Mothers’ experiences of care

The mothers in the present study reported a high level of satisfaction with providers’ skills, interpersonal relationships, and the overall care provided to them. However, they also expressed dissatisfaction with a few providers who use bad language. Similar findings were reported in the review of the literature that identified 24 studies in LMICs which showed that mothers’ rating of their satisfaction with care was high (75%) in the intervention area [30]. However, it was questioned whether this high satisfaction with care reported in the LMICs reflects good quality of care [30]. Studies have also shown paradoxical reports from mothers who report high satisfaction with care and at the same time complain of abuse and neglect by HCPs [30]. In Malawi, mothers reported high satisfaction because they did not know what to expect [31]. Similar to our baseline findings, mothers reported high satisfaction with care where PPC was poorly provided. We believe that the increased awareness about PPC after the intervention reported in the present study places mothers in a better position to describe sensibly their experiences of care and this increases the confidence that can be placed in the findings.

The improved quality may have ultimately increased mothers’ awareness and urge to seek good-quality PPC for themselves and their newborns. Srivastava et al. [30] claim that professional confidence, communication skills, competence, respect, and dignity are determinants for mothers’ satisfaction and influence their decisions to seek care. The mothers’ higher attraction to hospitals and health centres compared to dispensaries in the present study can be related to good quality of care at the former institutions, which triggers positive experiences of mothers and spreading of information about the improved quality, eventually leading to overcrowding at these institutions. When institutions are overwhelmed with clients the quality of care is, however, threatened [7], therefore the improvement of quality of PPC should happen equally across institutions. Supervision and accountability should ensure quality improvement and equal distribution of mothers/newborns at all levels of institutions.

### Strengths and limitations of the study

The main strengths of this study are the use of mixed methods and the general population sample. The research team was composed of experts with various professional backgrounds and nationalities and with substantial experience in research in different LMICs, which allowed for vivid discussions of alternative strategies and interpretations.

The limited number of PPC consultations observed during baseline hinders statistical testing of the difference between the IG and CG and thus, we describe the changes seen and perceived and do not test the effect of the intervention.

The outcome of the involvement of external experts to provide comments on the definition of quality of PPC was limited and should be seen as the start for further experts’ discussion. The response rate was low and perhaps the method of approaching them, with emails and phone calls, discouraged their participation. Face-to-face discussions might have been better for holding experts together and reaching consensus.

## Conclusions

The collegial facilitation intervention contributed to improved quality of PPC, HCP knowledge and professional confidence, awareness of PPC among mothers, and increased PPC attendance.

For refinement of this approach, collegial facilitation interventions are recommended in other settings to strengthen and scale up PPC. The work initiated here to define quality of PPC needs further development.

## Paper context

Postpartum care (PPC) is neglected despite the evidence that mothers/newborns die in the first weeks after childbirth particularly in sub-Saharan Africa. We conducted a study to evaluate the outcomes of a participatory facilitation intervention to improve PPC by assessing healthcare providers’ (HCPs’) knowledge, attitude, and perceptions towards PPC. Our findings suggest the need for using a participatory facilitation interventions to increase attendance and quality of PPC in institutions and for revising the definition of quality of care to prioritize care.

## Appendices

### Appendix 1. Frequency of observations in Ilala district after intervention.

**Table:**

	Performed	Not performed	% agreement /kappa value	
	*n* (%)	*n* (%)	%	*k*
**Getting ready**				
Greets the woman/companion respectfully and introduces her/himself	207 (97.2)	6 (2.8)	96.7	0.93
Encourages accompanying person to join if mother wishes	36 (16.9)	177 (83.1)	66.7	0.57
Tells the woman what is going to be done	152 (71.4)	61 (28.6)	96.7	0.93
Encourages the woman/partner to express her/their health concerns	132 (62.0)	81 (38.0)	88.3	0.81
**History**				
Asks the woman how she is feeling during the current postpartum period	143 (67.1)	70 (32.9)	96.7	0.93
Asks the mother whether her or the baby has had any problems since birth	176 (82.6)	37 (17.4)	100	1
Asks the woman how the baby is breastfeeding during the current period	173 (81.2)	40 (18.8)	100	1
Checks the woman’s record or asks for relevant history about her baby’s birth (date, mode, complications)	165 (77.5)	48 (22.5)	96.7	0.93
Checks the woman’s record or asks for result of her RPR test	73 (34.3)	140 (65.7)	96.7	0.93
Checks the woman’s record or asks for result of her HIV test	67 (31.5)	146 (68.5)	58.3	0.49
Checks for the mother’s prevention of mother to child transmission of HIV (PMTCT) records: C.D.4 count, stage of disease	38 (17.8)	175 (82.2)	86.7	0.93
Checks the woman’s record or asks for record of her tetanus toxoid (T.T) for the mother: T.T.1, T.T.2, T.T.3, T.T.4, T.T.5	145 (68.1)	68 (31.9)	96.7	0.93
Checks the woman’s record or asks for the records of oral poliovirus vaccine ( OPV), Bacillus Calmette–Guérin (BCG), and diphtheria, pertussis, and tetanus (DPT)-Hepatitis B+*Haemophilus influenzae* (Hib) (Penta valent)	137 (64.3)	76 (35.7)	95	0.90
Checks for drugs in use such as azidothymidine (AZT), Vitamin A	128 (60.1)	85 (39.9)	96.7	0.93
**Physical examination: mother**				
Observes the woman’s general appearance	162 (76.1)	51 (23.9)	100	1
Uses antiseptic hand rub or washes hands thoroughly	89 (41.8)	124 (58.2)	91.7	0.86
Takes her vital signs	79 (37.1)	134 (62.9)	100	1
Checks her conjunctiva for pallor	125 (58.7)	88 (41.3)	96.6	0.93
Examines her breasts	120 (56.3)	93 (43.7)	96.7	0.93
Palpates uterus for size, firmness, and tenderness	110 (51.6)	103 (48.4)	55	0.47
Puts gloves on, examines perineum for amount of lochia, condition of any tears or episiotomy, hemorrhoids, or other lesions	108 (50.7)	105 (49.3)	76.7	0.64
Assesses bladder function	33 (15.5)	180 (84.5)	88.3	0.80
Performs infection prevention procedures	48 (22.5)	165 (77.5)	85	0.79
**Physical examination: baby**				
Checks the baby’s color	164 (77.0)	49 (23.0)	100	1
Checks the baby’s breathing	177 (83.1)	36 (16.9)	100	1
Checks the baby is able to breastfeed	177 (83.1)	36 (16.9)	100	1
Checks baby’s ability to play/move	110 (51.6)	103 (48.4)	98.3	0.97
Checks the baby’s temperature	138 (64.8)	75 (35.2)	100	1
Weighs the baby	163 (76.5)	50 (23.5)	100	1
Examines from head to toe, checking for abnormalities	143 (67.1)	70 (32.9)	81.7	0.69
**Care of women’s psychological health**				
Assesses for irritability, tearfulness, and lability of mood	118 (55.4)	95 (44.6)	53.3	0.52
Asks about available social support system during postnatal period	139 (65.3)	74 (34.7)	55	0.47
Assesses for past or present history for mental health	11 (5.2)	202 (94.8)	63.3	0.53
Assesses for family history of perinatal mental disorder	2 (0.9)	211 (99.1)	56.7	0.55
Asks about partner’s relationship and misunderstandings	122 (57.3)	91 (42.7)	80	0.71
**Care provision: mother**				
Identifies problems based on history and examination, and acts accordingly (note: 2 observations missing)	89 (42.2)	122 (57.8)	75	0.63
Provides counseling about complication readiness, including danger signs	171 (80.3)	42 (19.7)	51.7	0.51
Provides counseling about nutrition, including iron supplementation	151 (70.9)	62 (29.1)	85	0.79
Provides counseling about rest and sleep	134 (62.9)	79 (37.1)	95	0.90
Provides counseling about hygiene (herself and the baby)	110 (51.6)	103 (48.4)	53.3	0.52
Provides counseling about malaria prevention (use of Insecticide-Treated Nets (ITN)) and helminthes	53 (24.9)	160 (75.1)	95	0.90
Provides counseling about safer sex and sexually transmitted infections (including HIV)	58 (27.2)	155 (72.8)	93.3	0.87
Provides counseling about sexuality and sexual practice resumption	103 (48.4)	110 (51.6)	100	1
Provides counseling on voluntary testing of HIV (if not done before)	46 (21.6)	167 (78.4)	91.7	0.85
Provides immunization and preventive therapy (iron, vitamin A, TT)	160 (75.1)	53 (24.9)	96.7	0.93
Provides counseling about family planning and methods of choice	141 (66.2)	72 (33.8)	100	1
Probes the woman/support person about experiences of or exposure to domestic violence	74 (34.7)	139 (65.3)	93.3	0.87
**Care provision: baby**				
Observes and counsels on breastfeeding	166 (77.9)	47 (22.1)	96.7	0.93
Insists on importance of exclusive breastfeeding or alternative feeding	153 (71.8)	60 (28.2)	100	1
Provides counseling about danger signs in the newborn period	177 (83.1)	36 (16.9)	96.7	0.93
Informs on what to do if the newborn experiences danger signs	177 (83.1)	36 (16.9)	100	1
Provides newborn immunization (if not done)	139 (65.3)	74 (34.7)	100	1
Encourages mother to ask questions about newborn and answers them properly	121 (56.8)	92 (43.2)	55	0.47
Informs mother/father date for next visit	205 (96.2)	8 (3.8)	100	1
Documents findings	176 (82.6)	37 (17.4)	100	1
**Communication skills**				
Continually informs mother of findings	139 (65.3)	74 (34.7)	100	1
Encourages mother to ask questions and responds accordingly	133 (62.4)	80 (37.6)	61.7	0.46
Makes woman/support person feel at ease	162 (76.1)	51 (23.9)	98.3	0.97
Verbal and non-verbal communication indicates interest	207 (97.2)	6 (2.8)	98.3	0.97

### Appendix 2. Items identified by experts as being required for PPC quality to be ‘Good’ or ‘Excellent’.

**Table:**

***Items (n = 46) required for ‘Good quality of PPC’***
Greets the mother/companion respectfully and introduces her/himself
Tells the mother what is going to be done
Encourages the mother/partner to express her/their health
Asks the mother how she is feeling during the current postpartum period
Asks the mother whether her or the baby has had any problems since birth
Asks the mother how the baby is breastfeeding during the current period
Checks the mother’s record or asks for relevant history about her baby’s birth (date, mode, complications)
Checks the mother’s record or asks for result of her RPR test
Checks the mother’s record or asks for result of her HIV test
Checks for the mother’s PMTCT records: cluster of differentiation 4 (CD 4) count, stage of disease
Checks the mother’s record or asks for a record of her tetanus toxoid (T.T): T.T.1, T.T.2, T.T.3, T.T.4, T.T.5
Checks the mother’s record or asks for the records of her OPV, BCG and D.P.T-Hep.B+Hib (Penta valent)
Checks for drugs in use such as AZT, Vitamin A
Observes the mother’s general appearance
Uses antiseptic hand rub or washes hands thoroughly
Takes her vital signs
Checks her conjunctiva for pallor
Examines her breasts
Palpates uterus for size, firmness, and tenderness
Puts gloves on, examines perineum for amount of lochia, condition of any tears or episiotomy, haemorrhoids, or other lesions
Assesses bladder function
Performs infection prevention procedures
Checks the baby’s color
Checks the baby’s breathing
Checks the baby is able to breastfeed
Checks the baby’s temperature
Weighs the baby
Examines from head to toe, checking for abnormalities
Assesses for irritability, tearfulness, and lability of mood
Assesses for past or present history of mental health
Asks about partner’s relationship and misunderstandings
Identifies problems based on history and examination, and acts accordingly
Provides counselling about hygiene (herself and the baby)
Provides counselling about malaria prevention (use of ITN) and helminths
Provides counselling on voluntary testing of HIV (if not done before)
Observes and counsels on breastfeeding
Insists on importance of exclusive breastfeeding or alternative feeding
Provides counselling about danger signs in the newborn period
Provides newborn immunization (if not done)
Encourages mother to ask questions about newborn and answers them properly
Informs mother/father of date for next visit
Documents findings
Continually informs mother of findings
Encourages mother to ask questions and responds accordingly
Makes mother/support person feel at ease
Verbal and non-verbal communication indicates interest
***Additional items (n = 13) required for ‘Excellent quality of PPC’***
Encourages accompanying person to join if mother wants
Checks baby’s ability to play/move
Asks about available social support system during postpartum period
Assesses for family history of perinatal mental disorder
Provides counselling about complication readiness, including danger signs
Provides counselling about nutrition, including iron supplementation
Provides counselling about rest and sleep
Provides counselling about safer sex and sexually transmitted infections (including HIV)
Provides counselling about sexuality and sexual practice resumption
Provides immunization and preventive therapy (iron, vitamin A, TT)
Provides counselling about family planning and methods of choice
Probes the mother/support person for experiences of or exposure to domestic violence
Informs on what to do if the newborn experiences danger signs
